# Fully digital workflow versus conventional methods for fabricating the Michigan appliance in patients with temporomandibular joint disorder. A randomized controlled clinical trial

**DOI:** 10.1186/s12903-026-08534-w

**Published:** 2026-05-13

**Authors:** Alshaimaa Ahmed Shabaan, Omar Ragab, Reham Ragab, Haitham Sharshar, Shaimaa Mohsen Refahee

**Affiliations:** 1 Oral & Maxillofacial Surgery Department, Faculty of Dentistry, Fayoum University, Fayoum, Egypt; 2 Specialist Periodontist and Implantologist, Private Practice, Sabah Al-Salem, Kuwait; 3 Nile of Hope hospital, Alexandria, Egypt; 4 Perio-implant researcher, Founder of digital dentistry schoology, Cairo, Egypt

**Keywords:** Temporomandibular Disorders, Occlusal Splints, Computer-Aided Design,3D Printing, digital workflow

## Abstract

**Objective:**

Fabrication of occlusal stabilization splints using fully digital workflow is feasible with several advantages over conventional workflow, including easy accessibility, time efficiency, and high accuracy in splint quality. Accordingly, the study’s aimed to evaluate the effect of fully digital fabricated occlusal splints on patient-centered outcomes compared to traditionally manufactured splints.

**Materials and methods:**

This is a prospective, randomized, blind study included 70 participants (35 in each group) with temporomandibular disorder (TMD) for which Michigan occlusal splint therapy was indicated. Group I participants received hard acrylic Michigan- splint fabricated using a fully digital workflow; while Group II participants received an identical Michigan splint fabricated using the conventional workflow. Pain intensity, maximum mouth opening (MMO), and oral health–related quality of life were assessed preoperatively and at 1, 3, and 6 months after the splint delivery.

**Results:**

Group I demonstrated a significantly lower pain scores and greater MMO compared to Group II (*p* ≤ 0.001) at the 1- and 3-months assessments. By the 6-months follow-up, pain scores were no longer significantly different between groups (*p* = 0.177). In contrast, the advantage for Group I in MMO remained statistically significant in 6 months (*p* = 0.001). Regarding to OHIP-14 total score, Group I reported better scores at all follow-up points (*p* ≤ 0.001). Additionally, the digital workflow significantly reduced treatment time (*p* < 0.001) and number of clinical visits(*p* < 0.001).

**Conclusion:**

A fully digital workflow for fabricating Michigan stabilization splints offers significant advantages in treatment efficiency and early clinical outcomes. However, both digital and conventional workflows demonstrated comparable long-term effectiveness in pain control.

**Supplementary Information:**

The online version contains supplementary material available at 10.1186/s12903-026-08534-w.

## Introduction

Temporomandibular disorders (TMD) are a group of conditions impacting the temporomandibular joint (TMJ) and associated structures. Temporomandibular disorders affect between 9.8% and 74% of different populations [1, 2]. It has been significantly more common in women than in men [3–5]. The clinical manifestations of TMD typically include pain in the joint and surrounding muscles, dysfunction, and a range of mechanical symptoms such as clicking and limited jaw movement [1]. The treatment of TMD includes a variety of approaches aimed at alleviating pain, improving jaw function, and addressing underlying causes. Given the complexity of TMD and the involvement of physical, psychological, and sometimes systemic factors, a multimodal strategy is typically employed [1, 6]. 

First-line treatment options for TMD commonly include conservative methods. Pharmacological therapies are frequently recommended, often alongside splint therapy, which is utilized to protect the teeth, reduce jaw clenching, and alleviate muscle strain [7–10]. The Michigan splint has been widely recognized as an effective therapeutic device for managing TMD. It is indicated for various conditions, including TMJ disorders, muscular pain, trauma from occlusion, and other aspects of the masticatory system. It can be used as an initial conservative treatment option and may be combined with other therapeutic modalities such as physical therapy. Research indicates that the Michigan splint can lead to significant improvements in symptoms associated with TMD, such as pain reduction, enhanced mandibular mobility, and better neuromuscular harmony. Studies suggest that this type of splint not only aids in relieving joint pain but can also facilitate better muscle relaxation, contributing to the overall efficacy of TMD management [11–13].

The fabrication of occlusal splints has evolved significantly, incorporating both traditional and modern digital methodologies. The primary methods of fabrication can be categorized into conventional techniques, CAD/CAM milling, and 3D printing [14].

Traditional methods of occlusal splint fabrication typically involve the use of vacuum-formed thermoplastic materials. The process usually includes several steps: obtaining a dental impression of the patient’s maxillary and mandibular arches, creating a working cast, and subsequently forming the splint by layering the material using the thermoforming method. This approach, while effective, often requires a considerable amount of time and may be subject to human error during the impression, jaw relation recording, and fabrication stages [14–16].

With CAD/CAM technologies, the fabrication of occlusal splints has transitioned to more automated processes. Using CAD, a digital model of the patient’s dental architecture is created, which can then be milled from solid blocks of biocompatible materials such as polycarbonate or PMMA. This subtractive method allows for a high degree of precision and reproducibility, significantly reducing error margins associated with manual methods. Studies have demonstrated that CAD/CAM-milled splints provide superior fit and performance compared to conventional methods, particularly in terms of structural integrity and wear resistance when subjected to occlusal forces. Moreover, three-dimensional (3D) printing has emerged as a revolutionary technique in the dental field, offering a new avenue for the rapid production of occlusal splints [14, 15, 17] The digital face bow is another technology used in prosthodontics to dynamically and accurately record a patient’s jaw relation.

Sun et al. [18], conducted a study to present a completely digital workflow for the fabrication of occlusal stabilization splints using CAD/CAM systems and a digital face bow based on optical sensor technology. They concluded that the fabrication of occlusal stabilization splints using a fully digital workflow is feasible with several advantages over traditional impression-based methods, including easy accessibility, time efficiency, high accuracy in splint quality, and the ability to easily produce duplicates.

The ongoing research is vital to address concerns regarding the mechanical properties of 3D-printed materials, which may still present challenges in terms of durability compared to those created with traditional acrylics. Moreover, a significant gap in knowledge exists regarding patient-centered outcomes; there is currently no research addressing whether these technical advantages ultimately impact patient comfort, satisfaction, or treatment efficacy. Accordingly, the aim of the current study was to evaluate the effect of fully digital fabricated occlusal splints on patient-centered outcomes compared to traditionally manufactured splints.

## Material & methods

### Study setting

The study was conducted in the Oral and Maxillofacial Surgery Department at the Faculty of Dentistry, Fayoum University, during the period spanning September 2023 to September 2025. Ethical approval was obtained from the ethics committee of Fayoum University (EC2356) in June 2023. The study’s protocol was registered on ClinicalTrials.org (NCT05959174). All patients gave informed consent for the scientific use of their clinical data and images before the study began. The study was conducted in compliance with the Declaration of Helsinki [19]and reported following the CONSORT guidelines 2025 [20].

### Study design and randomization

This is a prospective, randomized, blinded clinical trial with an allocation ratio of (1:1). The randomization sequence was an unstratified random block of sizes 2, 4, and 6 to confirm balance in the patients’ number allocated to each group. An investigator prepared a block randomization number list without clinical involvement in the trial. The outcome assessor and data analyzer were blinded to the group allocation.

### Sample size estimation

Based on data from A. H. Elkhadem and R. H. Hossameldin study [21], a sample size was calculated using G*Power 3.1.9.4. It was performed with 80% power, α = 0.05, and an effect size of 1.36. This analysis indicated a requirement of 32 participants. To compensate for potential dropouts, the study included 70 participants (35 per group).

### Participants

This study included 70 participants with temporomandibular disorder (TMD) for which Michigan occlusal splint therapy was clinically indicated. Patients were eligible for inclusion if they met all of the following criteria: (1) age ≥ 18 years; (2) presence of temporomandibular disorder (TMD) for which occlusal splint therapy was clinically indicated; (3) sufficient natural dentition to allow adequate retention and function of an occlusal splint; and (4) presence of TMD-related symptoms such as orofacial pain, jaw discomfort, muscle tenderness, or functional limitation. Patients were excluded if they met any of the following criteria: (1) presence of systemic musculoskeletal, neurological, or rheumatologic conditions affecting pain perception or mandibular function; (2) history of maxillofacial trauma, orthognathic surgery, or temporomandibular joint surgery within the preceding 12 months; (3) current use of an occlusal splint or history of splint therapy within the previous 6 months; (4) severe malocclusion, extensive tooth loss, or unstable occlusion preventing accurate splint fabrication; (5) active oral infection, untreated periodontal disease, or other oral conditions interfering with splint use; (6) pregnancy or lactation; (7) use of medications known to significantly influence pain perception; or (8) psychiatric or cognitive disorders that could compromise compliance or reliable reporting of patient-centered outcomes. Eligible participants were prospectively enrolled and randomly assigned to receive a maxillary full-coverage hard acrylic occlusal stabilization splint fabricated via one of two distinct manufacturing workflows: Group I (Digital group) participants in this group received a full-coverage hard acrylic Michigan- splint fabricated using a fully digital workflow; Group II (Conventional Group) participants in this group received an identical full-coverage hard acrylic Michigan splint fabricated using the conventional analog workflow.

### Intervention

Participants received a maxillary full-coverage hard acrylic Michigan splint fabricated via one of two distinct manufacturing workflows. The splint was standardized across both groups as it had a uniformly flat occlusal plane, simultaneous bilateral contacts in the therapeutic jaw position, and canine-guided excursive movements. The intervention differed solely in the method of data acquisition, design, and fabrication.

### Group I: fully digital workflow (Additional file 1,2,3,4)

#### Digital data acquisition

A high-resolution intraoral scanner (Trios 3, 3Shape, Copenhagen, Denmark) was used to capture digital impressions of the maxillary and mandibular arches. Data were exported as Standard Tessellation Language (STL) files. In addition, a digital facebow system (Zebris JMA system, Zebris Medical GmbH, Germany) employing optical sensor technology was utilized to transfer the jaw relation. Following a 30-minute of muscle deprogramming period using a cotton roll, the system’s sensors were affixed to the participant’s head and mandibular teeth. Participants performed a series of habitual opening/closing, protrusive, and lateral movements to record individual condylar kinematics and the envelope of motion.

**Fig. 1:**
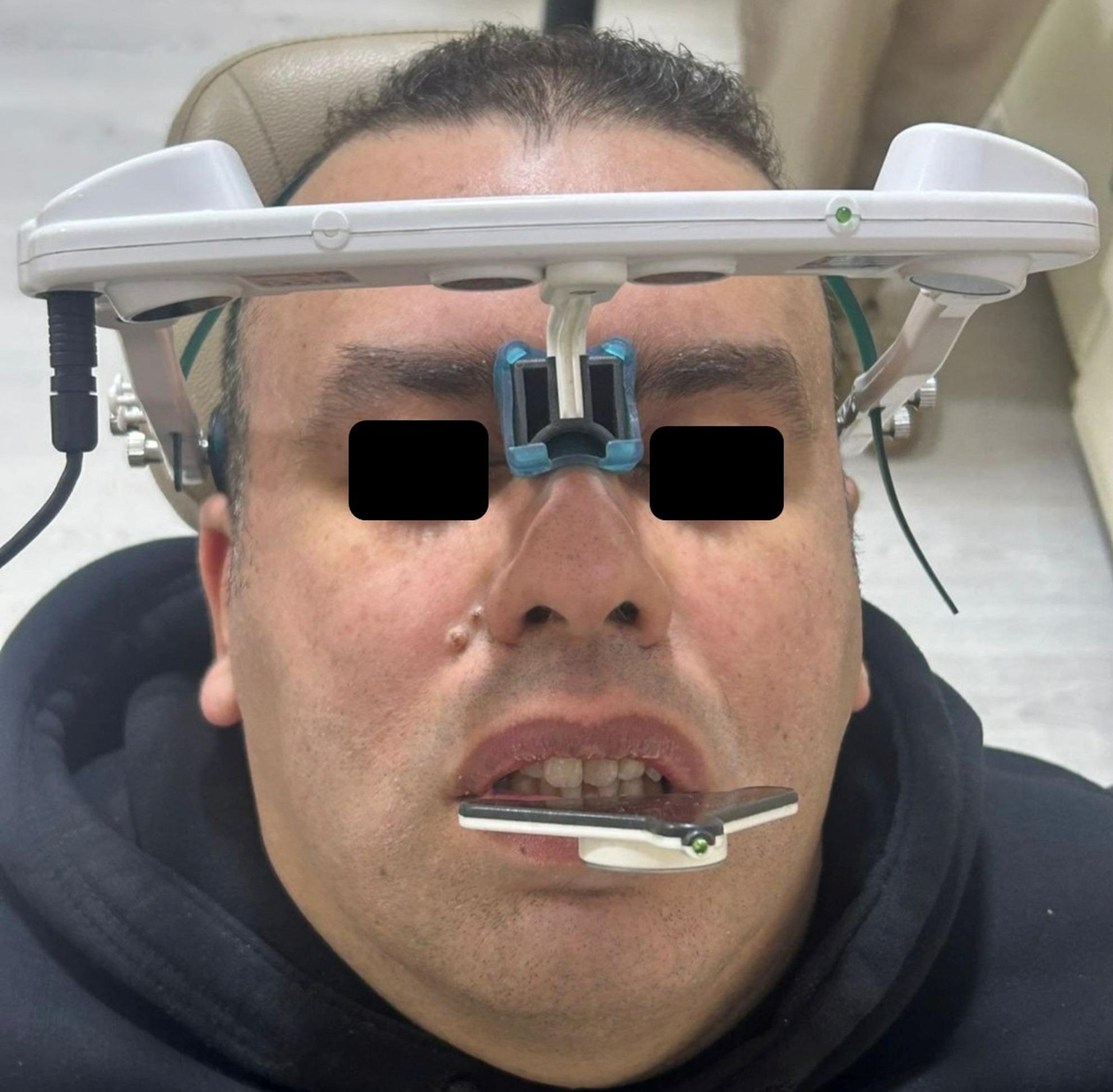
Jaw tracking capturing patient specific mandibular motion within a complete digital workflow

Within the recorded movement range, a reproducible centric relation position was identified. The vertical dimension of occlusion (VDO) was then virtually increased by 2.0 mm, referenced from the central fossae of the second molars, to establish the definitive jaw relation for splint design (Figure.1).

#### Computer- Aided Design (CAD)

The STL file of digital models and recorded mandibular movement trajectories were imported into CAD software (exocad DentalCAD 3.0, exocad GmbH, Darmstadt, Germany). The splint was designed virtually on the maxillary arch. The software’s virtual articulator function, driven by the participant-specific movement data, was used to perform a comprehensive virtual occlusal adjustment. This process ensured uniform centric stops and refined anterior guidance to provide smooth posterior disclusion during all excursive movements.

#### Computer- Aided Manufacturing (CAM-Additive Manufacturing)

The final design file was prepared for additive manufacturing with support structures and exported. The occlusal splint was fabricated using a digital light processing (DLP) 3D printer and a certified biocompatible Class IIa photopolymer resin specifically indicated for long-term occlusal splints. Post-processing included rinsing in isopropyl alcohol, removal of supports, and final polymerization in a dedicated UV-light curing unit according to the manufacturer’s protocol (Table 1).

**Table 1:** Summary of materials, printing parameters, and post-processing protocols for digital splint fabrication using LCD-based vat photopolymerization

Category	Key Specifications
Printer	Anycubic Photon Mono 2, MSLA, 405 nm UV
Resin	IFUN3D #3162, 75D hardness, ISO 10,993 compliant
Design SW	exocad DentalCAD v3.3, mandibular full-arch, 2.0 mm thickness
Orientation	Vertical (90°), no supports on intaglio surface
Layer thickness	100 μm
Exposure	Bottom: 35s, Normal: 2.5s
Post-curing	45 min at 405 nm + glycerin (final 10 min)
Cleaning	99% IPA, 10 min (two-stage)
Finishing	Carbide bur → Sof-Lex → rubber → pumice (water cooling)
Storage pre-test	Distilled water, 37 °C, 24 h

### Group II: conventional analog workflow

#### Analog data acquisition

A full-arch conventional impressions were made using vinyl polysiloxane material (Elite HD+, Zhermack, Badia Polesine, Italy) and poured in Type IV dental stone to create definitive casts. A centric relation record at the prescribed vertical dimension (increased by approximately 2.0 mm) was captured using a fast-setting silicone registration material (Futar D, Kettenbach GmbH, Eschenburg, Germany).

#### Manual design and fabrication

The mandibular cast was mounted using the inter-occlusal record. A stone model of the maxillary arch was prepared and duplicated. A sheet of clear, hard, 3.0-mm thick polymethyl methacrylate (PMMA) for vacuum forming was heated and vacuum-formed over the duplicate model using a standard vacuum-forming machine. The adapted acrylic sheet was trimmed to the desired outline, and the occlusal surface was lightly polished. The occlusion was not pre-adjusted in the laboratory and required full clinical adjustment at delivery.

#### Standardized delivery and adjustment

All splints were delivered by a single clinician. The internal adaptation and peripheral extension were checked and adjusted as needed for both groups. Occlusal contacts were verified using 20-micron articulating paper (Bausch Articulating Paper, Bausch GmbH, Köln, Germany).

For Group I, occlusal adjustment was performed to refine contacts; however, the time was anticipated to be reduced due to the pre-adjusted virtual occlusion. For Group II, comprehensive clinical occlusal adjustment was performed using articulating paper to establish even centric contacts and canine guidance, as the vacuum-forming process does not create a functional occlusal surface. All participants received identical, standardized verbal and written instructions regarding splint wear regimen (nocturnal use), maintenance, and care.

The total chairside adjustment time (in minutes) was recorded for each participant from splint construction to occlusion adjustment and delivery.

#### Outcome measurement

All participants were assessed preoperatively and re-evaluated at 1, 3, and 6 months after the splint delivery. The evaluated outcomes included pain intensity, maximum mouth opening (MMO), and oral health–related quality of life. Pain intensity was recorded using a 10-point visual analogue scale (VAS), where 0 revealed no pain and 10 represented the worst imaginable pain [22]. Maximum mouth opening was measured as the inter-incisal distance between the maxillary and mandibular central incisors [23]. Oral health–related quality of life was evaluated using the Oral Health Impact Profile-14 (OHIP-14) questionnaire, which comprises 14 items grouped into seven domains. Each item was scored on a five-point Likert scale (0 = never, 1 = hardly ever, 2 = occasionally, 3 = fairly often, and 4 = very often). The total OHIP-14 score was represented by summing all items, with possible scores ranging from 0 to 56; lower scores indicate better oral health–related quality of life, whereas higher scores reflect greater impairment [24].

#### Statistical analysis

All statistical analyses were performed using R software (version 4.5.0). Baseline continuous variables were assessed for normality using the Shapiro-Wilk test. Normally distributed baseline data are presented as mean ± standard deviation and were compared using independent t-tests. Non-normally distributed continuous variables are presented as median (min-max) and were compared using the Mann-Whitney U (Wilcoxon rank-sum) test. Categorical variables (e.g., Sex) were compared using Fisher’s exact test and are presented as counts (%).

Prior to longitudinal modeling, assumptions for standard parametric analysis were formally tested. Normality of residuals was checked via the Shapiro-Wilk test and Q-Q plots; homogeneity of variances was evaluated using Levene’s test; and homogeneity of regression slopes was tested via covariate interaction terms. Because the primary outcomes violated the assumptions of normality and homoscedasticity, because standard Repeated Measures ANCOVA was inappropriate, robust and non-parametric modeling strategies were employed to prevent Type I error inflation. Specifically, pain scores were analyzed across time points using robust ANCOVA (WRS2 package) adjusting for baseline pain. Maximum Mouth Opening (MMO) was evaluated using non-parametric rank-based methods for longitudinal data in factorial experiments (nparLD package). Furthermore, OHIP-14 total scores were analyzed utilizing linear models with heteroscedasticity-consistent standard errors (HC3 type, sandwich and lmtest packages) to robustly adjust for baseline values.For within-group changes from baseline, the Wilcoxon signed-rank test was utilized. For specific OHIP-14 subscales with highly limited response categories (2–3 unique differences), the Sign test (binomial test) was applied. To assess the clinical relevance of the within-group findings, non-parametric effect sizes were calculated (r=∣Z∣/ n) with values > 0.5 interpreted as large, clinically meaningful effects. Statistical significance was established at a two-sided α level of 0.05.

## Results

### Baseline characteristics and treatment protocols (figure.2)

At baseline, demographic and clinical characteristics were comparable between Group I and Group II, with no significant differences in age, gender distribution, pain scores, MMO, or OHIP-14 scores (Table 2). However, the treatment protocols differed significantly. Group I required a substantially shorter treatment duration (23.80 ± 2.63 min vs. 42.26 ± 3.85 min, *p* < 0.001) and fewer treatment visits (median 3 vs. 4, *p* < 0.001) than Group II.

**Fig. 2:**
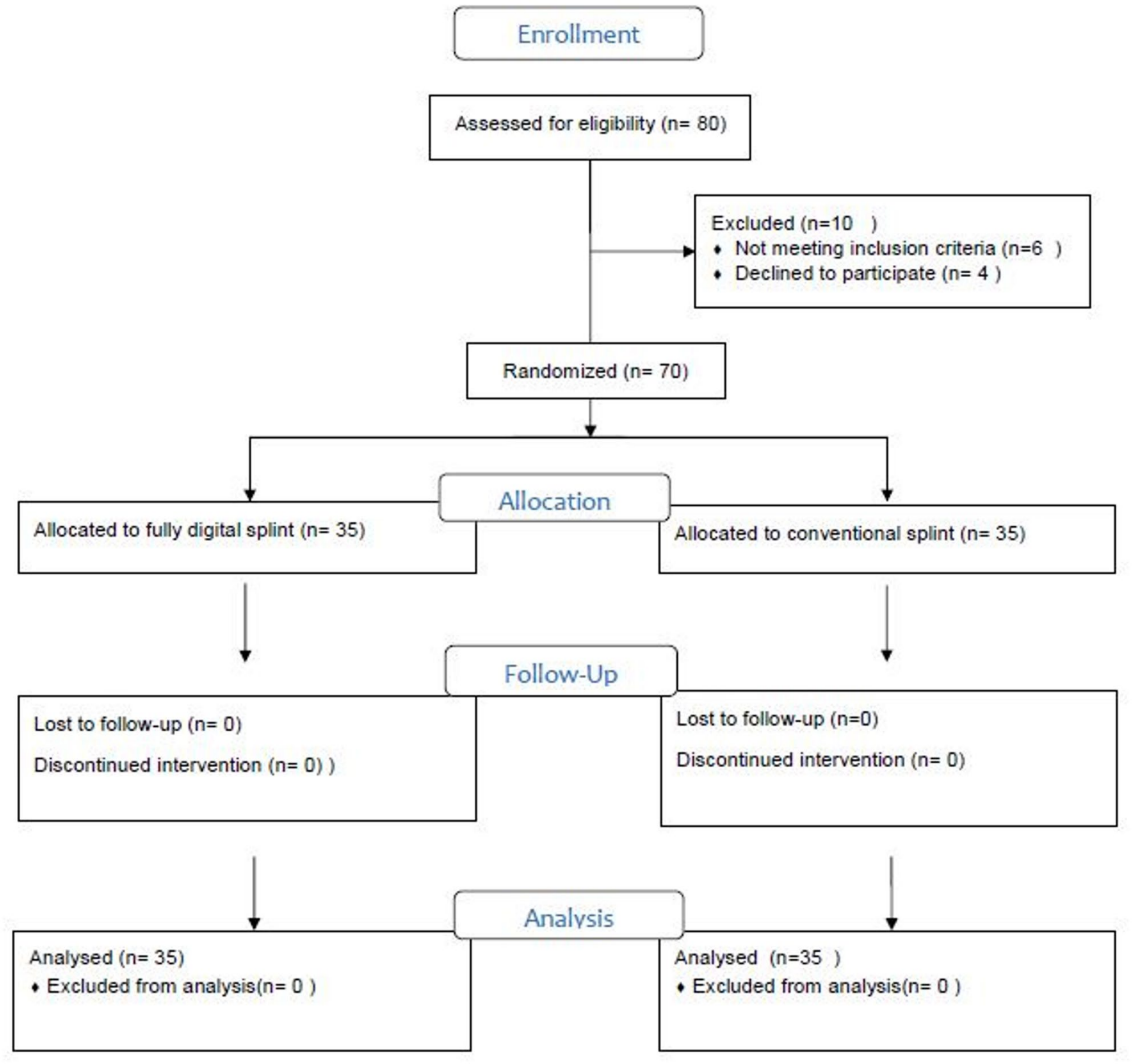
CONSORT flow chart

**Table 2:** Baseline Demographic and Clinical Characteristics by Treatment Group

Variable	Group I (*n* = 35)	Group II (*n* = 35)	*p* value
Gender: Female	27(77.1%)	28(80%)	1
Male	8(22.9%)	7(20)	
Age	32 (20–65)	34 (18–65)	0.387
Pain (at baseline)	7 (5–9)	7 (5–9)	0.33
MMO (at baseline)	30.37 ± 1.75	30.51 ± 1.32	0.707
OHIP-14 (at baseline)	33.69 ± 2.31	34.11 ± 2	0.409
Time (min)	23.80 ± 2.63	42.26 ± 3.85	< 0.001*
No. of Visits	3 (2–4)	4 (3–6)	< 0.001*

*p < 0.05

### Outcome measurements

Significant differences in clinical outcomes emerged during the follow-up period **(**Table 3) Group I demonstrated superior outcomes, with significantly lower pain scores and greater MMO compared to Group II (*p* ≤ 0.001) **)**, at the 1-month and 3-month assessments. By the 6-month follow-up, pain scores had converged and were no longer significantly different between groups (*p* = 0.177). In contrast, the advantage for Group I in MMO remained statistically significant in 6 months (*p* = 0.001).

**Table 3:** Comparison of Clinical Outcomes Between Groups at Each Follow-up Period

Variable	Group I	Group II	*p* value
Pain M1	2 (1–4)	4 (3–6)	< 0.001*
Pain M3	0 (0–2)	1 (0–2)	0.001*
Pain M6	0 (0–2)	0 (0–1)	0.177
MMO M1	33.54 ± 0.91	32.45 ± 0.99	< 0.001*
MMO M3	34.80 (33.40–37.80)	33.90 (32.20–36.30)	0.001*
MMO M6	34.8 (33.4–37.8)	33.9 (32.2–36.5)	0.003*
OHIP-14 M1	19 ± 1.61	22.46 ± 2.57	< 0.001*
OHIP-14 M3	8 (6–11)	11 (9–14)	< 0.001*
OHIP-14 M6	3 (0–6)	5 (1–7)	0.001*

*p < 0.05

OHIP-14 total scores improved significantly in both groups over time, but Group I consistently reported better scores at all follow-up points (1, 3, and 6 months; *p* ≤ 0.001). Analysis of OHIP-14 subscales revealed a dynamic pattern of treatment response **(**Table 4**).** While Group II reported higher baseline psychological discomfort, Group I showed superior early improvement in this domain by 1 month (*p* = 0.002). Group I also demonstrated significantly greater improvement in psychological disability at all post-baseline assessments (*p* < 0.001) and in social disability at 3 months (*p* < 0.001). Conversely, Group II maintained an advantage in lower handicap scores from baseline through the 6-month follow-up (*p* ≤ 0.007).

**Table 4:** Longitudinal Comparison of OHIP-14 Subscale Scores Between Treatment Groups

Variable	Time Point	Group I	Group II	*p* value
psychological discomfort	Baseline	3.0 (2.0–4.0)	4.0 (2.0–5.0)	< 0.001*^†^
	1 Month	2.0 (1.0–3.0)	1.0 (1.0–2.0)	0.002*
	3 Months	1.0 (0.0–1.0)	1.0 (0.0–1.0)	1
	6 Months	1.0 (0.0–1.0)	1.0 (0.0–1.0)	1
physical disability	Baseline	5.0 (3.0–6.0)	5.0 (4.0–6.0)	0.159^†^
	1 Month	2.0 (1.0–3.0)	3.0 (2.0–5.0)	< 0.001*
	3 Months	1.0 (1.0–3.0)	2.0 (1.0–3.0)	0.381
	6 Months	0.0 (0.0–2.0)	0.0 (0.0–1.0)	0.063
psychological disability	Baseline	6.0 (5.0–7.0)	7.0 (5.0–7.0)	0.292
	1 Month	3.0 (2.0–5.0)	5.0 (3.0–6.0)	< 0.001*^†^
	3 Months	1.0 (0.0–3.0)	2.0 (1.0–3.0)	< 0.001*
	6 Months	0.0 (0.0–1.0)	1.0 (0.0–2.0)	< 0.001*
social disability	Baseline	6.0 (5.0–7.0)	6.0 (5.0–6.0)	0.026*
	1 Month	5.0 (3.0–6.0)	5.0 (4.0–6.0)	0.159
	3 Months	2.0 (1.0–3.0)	3.0 (2.0–5.0)	< 0.001*
	6 Months	1.0 (0.0–1.0)	1.0 (0.0–1.0)	1
handicap	Baseline	2.0 (1.0–3.0)	1.0 (1.0–2.0)	0.001*
	1 Month	1.0 (0.0–2.0)	1.0 (0.0–2.0)	0.879
	3 Months	0.0 (0.0–1.0)	0.0 (0.0–2.0)	0.007*
	6 Months	0.0 (0.0–1.0)	0.0 (0.0–2.0)	0.005*

* *p*-value < 0.05 ; †: Wilcoxon rank sum test other p-value is Fisher Exact test

A longitudinal analysis of OHIP-14 scores, adjusted for baseline values using a robust model to account for heteroscedasticity, confirmed a significant group effect (B = 3.24, 95% CI [1.87, 4.61], *p* < 0.001), indicating consistently better outcomes for Group I. There was also a significant main effect of time (F = 373.21, *p* < 0.001) and a significant Group × Time interaction (F = 7.89, *p* < 0.001), demonstrating that the pattern of improvement differed between the two protocols. The model explained a substantial portion of the variance in outcomes (R²=0.92). Reliable longitudinal models for Pain and MMO could not be estimated due to violations of parametric assumptions; nonparametric results for these outcomes are presented in (Table 5).

**Table 5:** Results of Repeated Measures Analysis with Corrections for Assumption Violations

Outcome	Group Effect	Time Effect	Interaction	Baseline Effect	*R* squared	Violation Correction
OHIP-14	3.24 (1.87, 4.61)†	F = 373.21, *p* < 0.001	F = 7.89, *p* < 0.001	0.52 (0.39, 0.65)†	0.92	HC3 robust SE
Pain	Not estimable*	Not estimable*	Not estimable*	Not estimable*	--	Nonparametric alternative
MMO	Not estimable*	Not estimable*	Not estimable*	Not estimable*	--	Rank-based method

† Values shown as Estimate (95% CI), * Could not be reliably estimated due to assumption violations

### Within-group changes from baseline

Within-group analyses confirmed that both treatments yielded significant and clinically meaningful improvements from baseline across all outcome measures, with large effect sizes (*r* > 0.8) **(**Table 6**).** While effective for both groups, the magnitude of improvement frequently differed. Group I consistently demonstrated superior short-term gains, with greater median reductions in pain and greater improvements in both maximum mouth opening (MMO) and OHIP-14 total scores at the 1- and 3-month assessments. By the 6-month follow-up, the degree of improvement had converged for pain and OHIP-14 total scores, though Group I maintained a marginally larger gain in MMO. A nuanced analysis of OHIP-14 subscales further delineated these patterns **(**Table 7**)**, revealing that Group I achieved a more rapid and ultimately greater improvement in psychological disability, and a consistently larger reduction in perceived handicap. In contrast, both groups achieved similar, substantial long-term improvements in the domains of physical and social disability, despite differing trajectories.

**Table 6:** Within-Group Analysis for Pain, MMO, and OHIP-14 (Wilcoxon Signed-Rank Test)

Measurement	Group	Time point	Median Difference ^a^	IQR	*p*-value	Effect Size (*r*)
Pain	Group I	M1	-5	[-6, -4]	< 0.001*	0.88
		M3	-7	[-8, -6]	< 0.001*	0.87
		M6	-7	[-8, -6]	< 0.001*	0.87
	Group II	M1	-3	[-4, -2]	< 0.001*	0.85
		M3	-6	[-6, -5]	< 0.001*	0.88
		M6	-7	[-7.5, -6]	< 0.001*	0.88
MMO	Group I	M1	3.1	[2.05, 4.25]	< 0.001*	0.87
		M3	4.5	[3.55, 5.55]	< 0.001*	0.87
		M6	4.5	[3.4, 5.55]	< 0.001*	0.87
	Group II	M1	2.2	[0.85, 2.9]	< 0.001*	0.81
		M3	3.9	[2.55, 4.6]	< 0.001*	0.87
		M6	4	[2.55, 4.6]	< 0.001*	0.87
OHIP-14	Group I	M1	-15	[-16, -13]	< 0.001*	0.87
		M3	-25	[-26.5, -24]	< 0.001*	0.87
		M6	-30	[-32, -29]	< 0.001*	0.87
	Group II	M1	-12	[-13, -10]	< 0.001*	0.87
		M3	-23	[-25, -22]	< 0.001*	0.87
		M6	-30	[-31, -28]	< 0.001*	0.87

*p < 0.05 ; a: Median Difference = median time point – median baseline

**Table 7:** Within-Group Improvements in OHIP-14 Subscale Scores from Baseline

Variable	Group	Time Point	Median Difference	IQR	*p* value
psychological discomfort	Group I	M1	-1	[-2.0, -1.0]	< 0.001*†
		M3	-3	[-3.0, -2.0]	< 0.001*
		M6	-3	[-3.0, -2.0]	< 0.001*
	Group II	M1	-2	[-3.0, -2.0]	< 0.001*
		M3	-3	[-4.0, -3.0]	< 0.001*
		M6	-3	[-4.0, -3.0]	< 0.001*
physical disability	Group I	M1	-3	[-3.0, -2.0]	< 0.001*
		M3	-3	[-4.0, -2.0]	< 0.001*
		M6	-4	[-5.0, -3.0]	< 0.001*
	Group II	M1	-2	[-2.0, -1.0]	< 0.001*
		M3	-3	[-3.0, -3.0]	< 0.001*
		M6	-4	[-5.0, -4.0]	< 0.001*
psychological disability	Group I	M1	-4	[-4.0, -2.0]	< 0.001*
		M3	-6	[-6.0, -5.0]	< 0.001*
		M6	-6	[-7.0, -6.0]	< 0.001*
	Group II	M1	-2	[-3.0, -1.0]	< 0.001*
		M3	-4	[-5.0, -4.0]	< 0.001*
		M6	-6	[-6.0, -5.0]	< 0.001*
social disability	Group I	M1	-1	[-2.0, -1.0]	< 0.001*
		M3	-4	[-4.0, -3.0]	< 0.001*
		M6	-5	[-6.0, -4.0]	< 0.001*
	Group II	M1	-1	[-1.0, 0.0]	< 0.001*
		M3	-3	[-3.0, -2.0]	< 0.001*
		M6	-5	[-5.0, -4.0]	< 0.001*†
handicap	Group I	M1	-1	[-1.5, 0.0]	< 0.001*
		M3	-2	[-2.0, -1.5]	< 0.001*
		M6	-2	[-2.0, -1.5]	< 0.001*
	Group II	M1	-1	[-1.0, 0.0]	< 0.001*
		M3	-1	[-2.0, -0.5]	< 0.001*†
		M6	-1	[-2.0, -0.5]	< 0.001*†

*All p-values significant at < 0.001 level; Sign test used for variables with limited response categories

### Time course of treatment response

**Fig. 3:**
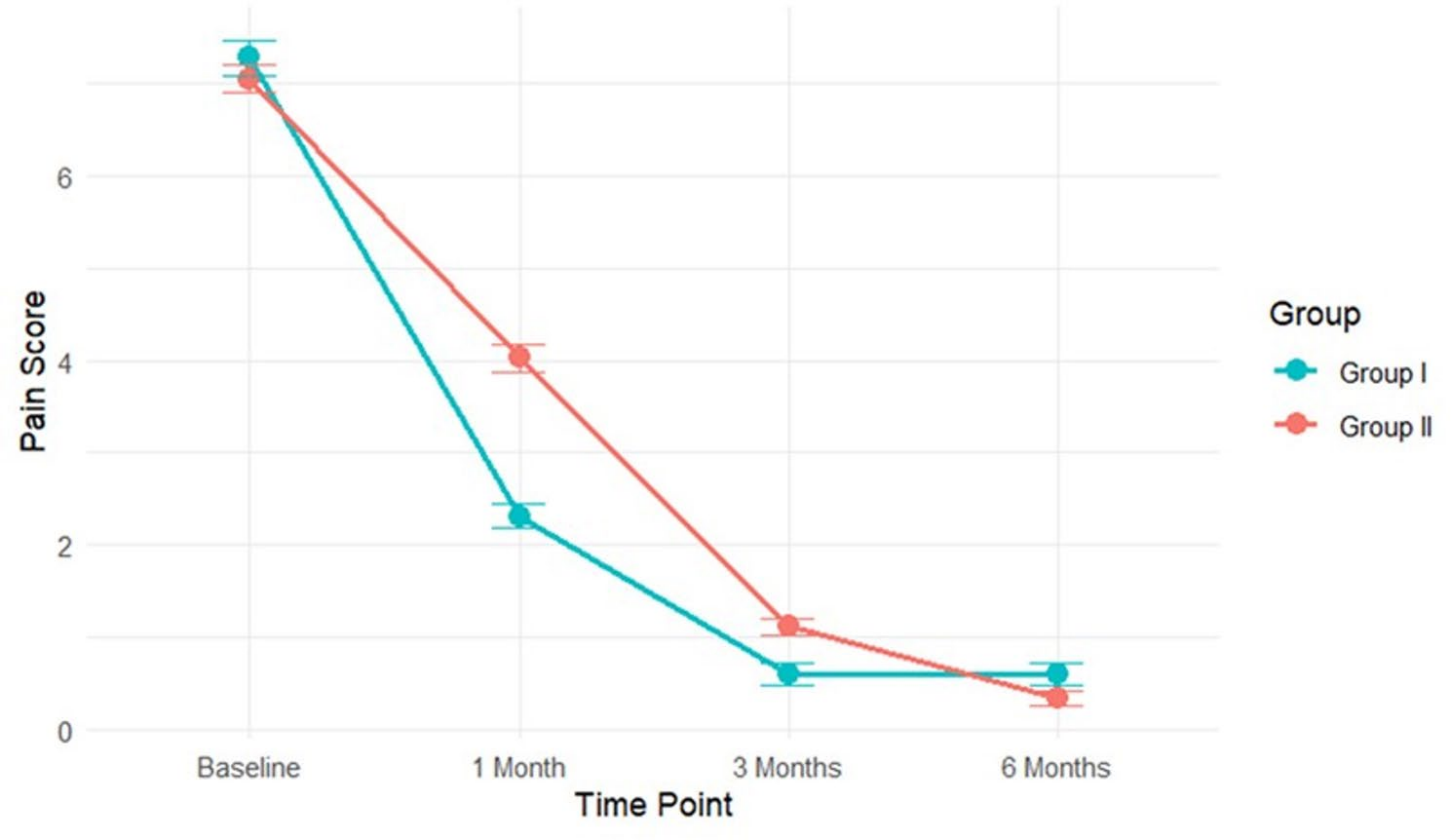
Changes in Pain Score Over Time

**Fig. 4:**
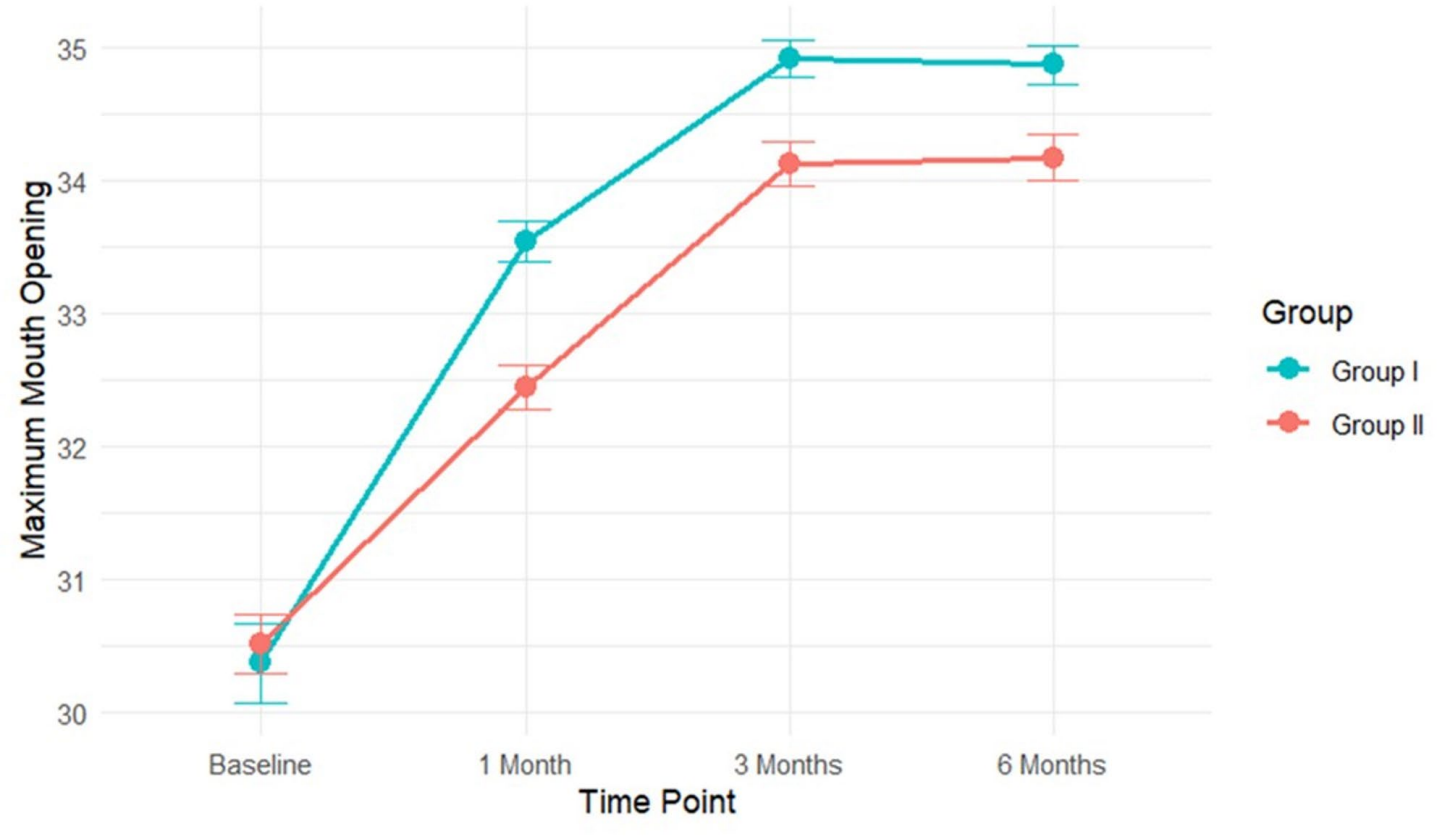
Changes in MMO Over Time

**Fig. 5:**
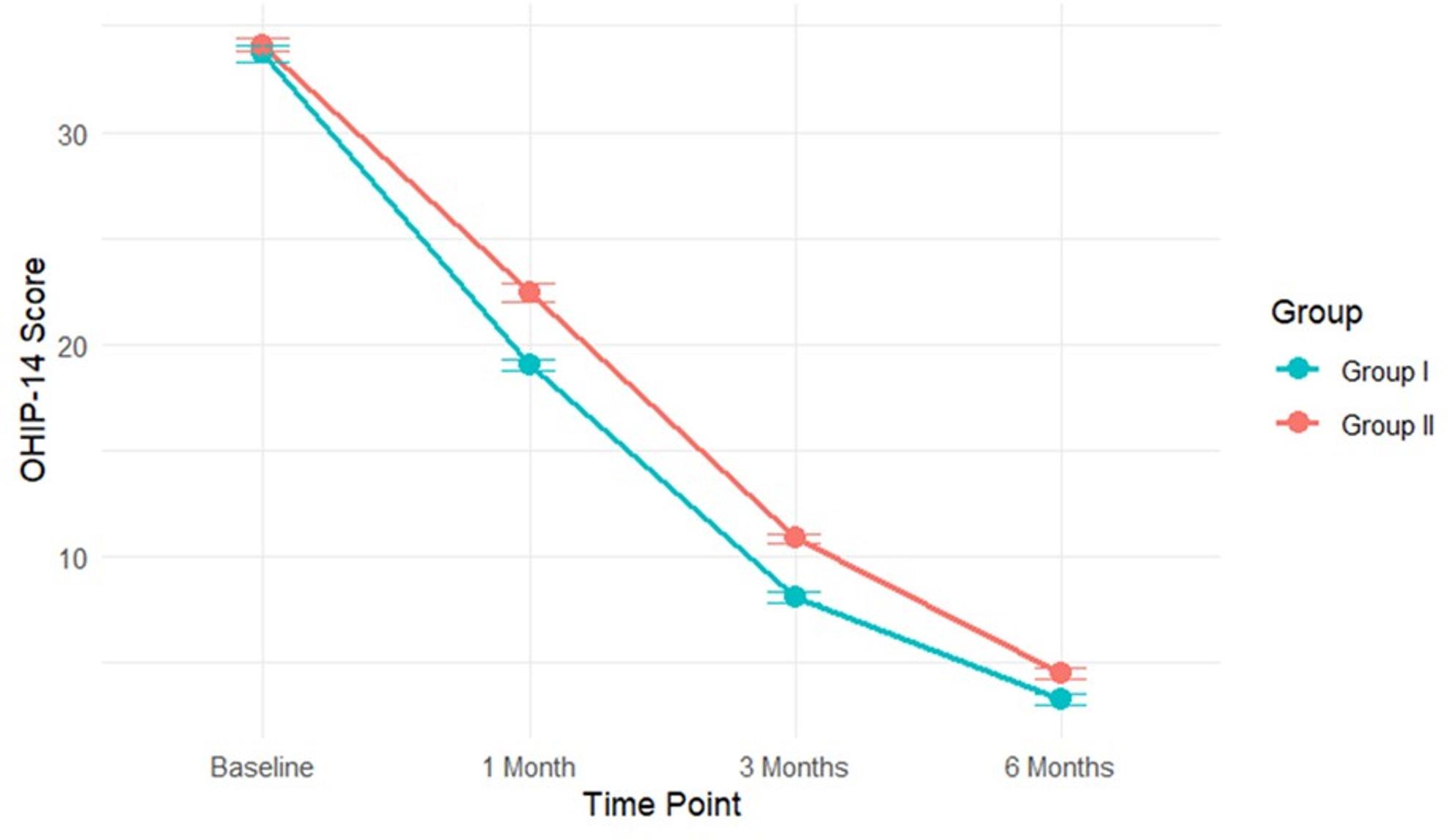
Changes in OHIP-14 score Over Time

Visual analysis of the outcome trajectories **(**Figs. 3, 4 and 5**)** illustrated the temporal patterns. Group I exhibited a steeper initial decline in pain and a more rapid increase in MMO during the first month, maintaining this advantage through the 3-month assessment. For OHIP-14 scores, Group I consistently maintained lower (better) scores throughout the entire follow-up period. The most rapid improvements for all outcomes occurred within the first month, with more gradual gains thereafter.

## Discussion

The integration of digital workflows in the management of TMDs provides significant advantages that enhance efficiency, precision, and patient satisfaction in dental care. Digital workflows utilize advanced technologies such as computer-aided design (CAD), 3D printing, and virtual patient modeling, revolutionizing traditional treatment protocols. The findings of the current study demonstrate that the fabrication method of a Michigan-type stabilization splint significantly influences both the clinical trajectory and the efficiency of TMD therapy. Although both techniques provided substantial therapeutic benefit, the fully digital workflow offered clinically meaningful advantages, including faster treatment delivery, superior early symptom relief, and greater improvements in functional jaw mobility and oral health-related quality of life, particularly within the critical initial months of therapy.

The advancement of digital workflows in dentistry has led to notable reductions in both treatment duration and the number of visits required. The results of the current study highlight that the treatment time for digital workflows has reduced significantly compared to conventional workflows, and that the median number of visits is reduced from four to three. Several interrelated factors contribute to these differences, including enhanced accuracy, efficiency in procedural execution, and patient-centric benefits. The decline in treatment duration can be primarily attributed to the digitization of impression techniques and the subsequent fabrication processes. Digital impression techniques using intraoral scanners facilitate a more straightforward procedure compared to traditional methods that require physical impressions, pouring, and sometimes remaking casts due to inaccuracies [25–27]. Studies have shown that digital adjustments and real-time monitoring significantly reduce the complexity involved in creating accurate occlusal relationships, thus trimming the treatment time considerably [26]. The ability to simulate and visualize outcomes before actual procedures further reduces the likelihood of adjustments during the treatment process, providing a smoother workflow and minimizing the need for additional visits. Moreover, the integration of digital tools streamlines communication between dental professionals and laboratories, which enhances overall workflow efficiency. For instance, the virtual transmission of designs allows for quicker fabrication of appliances and immediate adjustments based on pre-treatment simulations [18, 28]. These contrast sharply with conventional workflows, where intermediaries, such as physical model creation and laboratory communication, can delay treatment progression and extend appointment lengths.

The study showed a significant difference in clinical outcomes between the two treatment groups regarding pain scores and MMO during follow-up assessments. These clinical outcomes can be attributed to fundamental differences in precision, reproducibility, and biomechanical optimization afforded by the respective workflows.

The fully digital protocol leveraged high-resolution intraoral scanning, dynamic jaw registration via a digital facebow, and virtual articulation to design and pre-adjust the occlusal splint prior to fabrication. This approach minimizes cumulative errors associated with conventional impression-taking, cast pouring, and manual laboratory and clinical adjustments. Consequently, the digitally produced splint likely delivered a more accurate and physiologically appropriate occlusal scheme from the moment of delivery, promoting immediate neuromuscular deprogramming, optimal condylar positioning, and reduced articular and muscular strain. By the six-month follow-up, pain scores between the two groups had converged, suggesting that both modalities are ultimately capable of achieving comparable levels of pain control once the conventional splint is adequately adjusted and the patient adapts [18, 29–31].

In contrast, the persistent and statistically significant advantage in MMO retained by the digital group at six months indicates a lasting functional benefit beyond pain modulation. This finding likely reflects the superior biomechanical efficacy of a splint fabricated with patient-specific kinematic data. The precise guidance and occlusal contacts engineered into the digital splint may have promoted more favorable joint loading and muscular coordination during the critical early rehabilitative phase, leading to enhanced tissue adaptation and a greater final range of motion [18, 32].

Pre-adjusted virtual occlusion provides a mechanistic advantage by establishing a stable, centrically referenced occlusal target before any physical contacts are introduced. This approach decouples the initial motor learning phase from the trial-and-error adjustments inherent to conventional workflows. By simulating an optimal intercuspal position using digital occlusal analysis and virtual articulation, the clinician directs the jaw toward a prevalidated neuromuscular target, constraining the range of possible motor commands during early function [33–35]. This reduces unnecessary co-contractions and aberrant muscle activity, allowing the masseter and temporalis muscles to align with a balanced proprioceptive map from the outset [33]. Consequently, the central nervous system receives consistent afferent input from craniofacial proprioceptors configured around the virtual reference position, promoting smoother sensorimotor integration and faster recalibration toward symmetrical muscle activation [36]. Additionally, precise control of occlusal contacts minimizes initial interferences, limiting reflexive protective co-contractions and preserving postural stability during early functional tasks. Collectively, these mechanisms accelerate neuromuscular adaptation and shorten the time required to achieve a stable, balanced occlusal state [21, 36, 37].

The observed clinical advantages of the digital workflow may also be partly explained by differences in material properties and fabrication precision. Three-dimensionally printed splints typically exhibit superior surface smoothness and structural homogeneity compared to conventionally vacuum-formed polymethyl methacrylate (PMMA) appliances, which can be affected by air bubbles, thickness inconsistencies, and surface irregularities [16, 38]. A smoother intaglio surface reduces friction against the oral mucosa and may enhance proprioceptive feedback by providing more consistent tactile input to periodontal mechanoreceptors and mucosal receptors, potentially improving neuromuscular control and patient comfort during early wear [14, 16]. Furthermore, the digital workflow employed a digital facebow system, which uses optical sensor technology to dynamically record individual condylar kinematics, including habitual opening/closing, protrusive, and lateral movements. This kinematic accuracy allows for the transfer of patient-specific jaw relation data into the CAD software, enabling the design of a splint that is dynamically harmonized with the patient’s unique envelope of motion. In contrast, conventional methods rely on static centric relation records, which cannot capture individualized condylar pathways, potentially leading to occlusal interferences during excursive movements. The combination of precise material properties and kinematic accuracy thus provides a mechanistic basis for the digital workflow’s superior early outcomes [14, 35, 39].

The Oral Health Impact Profile-14 (OHIP-14) results provide a patient-centered perspective that extends beyond traditional clinical metrics. The finding that both groups experienced significant improvements in OHIP-14 total scores over time reinforces the fundamental therapeutic efficacy of the Michigan stabilization splint in alleviating the global burden of TMD, regardless of fabrication method [40–42].

However, the consistently superior OHIP-14 scores in digital group at all follow-up intervals indicate that the digital workflow confers a meaningful additive benefit to the patient’s lived experience of treatment. This advantage is particularly illuminated by subscale analysis, which reveals distinct psychosocial trajectories [43, 44].

The rapid and superior early improvement in psychological discomfort (e.g., feelings of tension, worry, or self-consciousness about the jaw condition) observed in digital group by one month likely stems directly from the speed and predictability of their clinical experience. A splint that fits precisely from delivery requires minimal adjustment, and provides faster pain relief can significantly reduce treatment-related anxiety and enhance a patient’s sense of control and optimism, leading to swift psychological benefits [45, 46].

The sustained and greater improvement in psychological disability in digital group across all follow-ups suggests a deeper, more enduring positive impact on mental well-being. This may be attributable to the cumulative effect of faster functional recovery and the reassurance provided by a highly precise, technologically advanced treatment process. Patients may perceive the digital approach as more modern and reliable, fostering greater confidence in the treatment outcome [18, 21].

The transient but significant advantage for digital group in social disability at three months may reflect a critical period where faster pain relief and functional improvement allowed patients to reintegrate into social and communal activities more quickly than their counterparts in the conventional group [44, 46].

Conversely, the consistent finding that conventional group reported lower scores in the *handicap* domain is clinically insightful. The handicap subscale measures the perceived inability to perform life roles or a sense that “life is less satisfying.” It is possible that patients undergoing the conventional, more time-intensive workflow may have developed lower initial expectations or a different internal benchmark for “success.” Their significant improvement from a painful baseline state, achieved through a more familiar, hands-on therapeutic process, might have resulted in a profoundly satisfying sense of gain, thereby reducing feelings of handicap despite slower initial progress. This highlights the complex interplay between treatment modality, patient expectations, and the subjective appraisal of life impact [42–44].

The observed difference in OHIP-14 total scores between groups exceeds the reported minimal clinically important difference of 3.5 points for TMD patients, indicating that the digital workflow’s advantage is not only statistically significant but also clinically meaningful.

Beyond clinical metrics, our findings suggest that the digital workflow’s efficiency itself contributed to the superior early outcomes. The streamlined process likely reduces treatment burden, enhancing patient perception and adherence. It is speculative that the 3D-printed appliances, such as superior surface smoothness and structural homogeneity, may have provided enhanced comfort and proprioceptive feedback, directly influencing patient-reported function and pain relief from the outset [18, 29].

Our findings regarding the technical feasibility and precision of the digital workflow are strongly supported by the foundational work of Sun et al. [18]. Their study presented a fully digital protocol similar to ours, utilizing intraoral scanning and a digital facebow for dynamic jaw registration, and concluded that the method was feasible and offered high accuracy. Our results confirm this feasibility in a controlled clinical trial setting and critically advance the evidence by demonstrating its clinical superiority. While Sun et al. focused on technical and laboratory parameters, our RCT provides direct evidence that this technical precision translates into superior early patient outcomes. Specifically, the steeper initial decline in pain and more rapid increase in MMO in our digital group can be directly attributed to the “pre-adjusted” occlusion achieved through virtual articulation.

The adoption of a fully digital workflow for occlusal splint fabrication requires consideration of cost, accessibility, and feasibility in routine practice. From a financial perspective, the digital workflow entails upfront investment in an intraoral scanner, CAD software, a 3D printer, and post-processing equipment. However, these initial costs may be offset over time by reduced chairside time, fewer clinical visits, and lower labor costs associated with digitization and automation. While per-unit material costs for 3D-printed resins can be higher than conventional acrylic, the overall efficiency gains and reproducibility may achieve cost-neutrality or savings in high-volume settings [16, 21, 47].

Accessibility remains a key consideration. In well-equipped dental clinics or academic institutions with sufficient patient volume, digital workflows are readily implementable and have been shown to improve patient comfort and clinical efficiency. Conversely, in small or resource-limited practices, the capital expenditure for hardware and software, along with staff training requirements, may pose barriers to adoption. In such settings, conventional methods remain a feasible and effective alternative, particularly given the comparable long-term pain control observed in this study [12, 15, 21].

Feasibility in routine practice is supported by the growing body of evidence demonstrating that digital splints can be integrated into standard clinical workflows without prolonging appointment times. With appropriate training and standardized protocols, digital fabrication is reproducible, reduces operator dependence, and enhances patient-centered outcomes. Therefore, while digital workflows are not yet universally accessible, they represent a practical and advantageous option for practices that can accommodate the initial investment [14, 16].

This study has several limitations. First, its single-center design and modest sample size, while adequate for this initial comparison, suggest that results should be replicated in larger, multi-center trials to enhance generalizability. Second, while outcome assessors were blinded, complete blinding of clinicians to the splint type during delivery and follow-up adjustments was not feasible, which is a common challenge in trials of medical devices. Third, the 6-month follow-up period is sufficient to assess initial efficacy but cannot determine long-term outcomes; future studies of 1–2 years are needed to evaluate treatment durability, sustained patient compliance, and cost-effectiveness. Finally, our findings are tied to the specific digital system used; results may vary with different hardware, software, or material combinations.

A further limitation of this study is the absence of objective functional parameters, such as surface electromyography (EMG) for masticatory muscle activity, standardized joint sound analysis, or kinematic tracking of mandibular movements. These objective measures could have provided mechanistic insight into why the digital workflow demonstrated superior early outcomes, particularly the sustained improvement in maximum mouth opening.

Future research should address these limitations through several key avenues. First, longitudinal studies with follow-up periods of 1–2 years are needed to confirm the durability of the observed advantages and to perform formal cost-effectiveness analyses, ideally integrating both subjective patient-reported outcomes and objective functional assessments to better understand how digital versus conventional splints influence TMD pathophysiology. Second, applying this protocol to broader patient populations would test its generalizability. Third, direct comparisons within digital workflows, specifically between additive and subtractive fabrication methods, are required to refine best practices and optimize resource allocation.

## Conclusions

Within the limitations of this study, a fully digital workflow for fabricating Michigan stabilization splints offers significant advantages in treatment efficiency and early clinical outcomes. It is associated with faster pain reduction, greater improvement in maximum mouth opening, and better oral health–related quality of life during the initial phases of therapy.

Both digital and conventional workflows demonstrated comparable long-term effectiveness in pain control. While the digital approach provides a more favorable early therapeutic trajectory and sustained functional benefits, it should be considered a clinically advantageous option primarily in the short term rather than an overall superior modality.

## Supplementary Information

Supplementary Material 1. Additional file 1: Showing dynamic jaw movement registration.

Supplementary Material 2. Additional file 2: Dynamic condyle tracking.

Supplementary Material 3. Additional file 3: A hard acrylic Michigan- splint fabricated using a fully digital workflow.

Supplementary Material 4. Additional file 4: Jaw tracking capturing patient specific mandibular motion within a complete digital workflow.

## Data Availability

The data used and/or analyzed during the current study are available from the corresponding author on reasonable request.

## Abbreviations

CAD: Computer-aided design
CAM: Computer- Aided Manufacturing
MMO: Maximum mouth opening
OHIP-14: The Oral Health Impact Profile-14
PMMA: Polymethyl methacrylate
STL: Standard Tessellation Language
TMD: Temporomandibular disorders
VAS: Visual analogue scale
VDO: Vertical dimension of occlusion
