# Supplementary material for: Fully digital workflow versus conventional methods for fabricating the Michigan appliance in patients with temporomandibular joint disorder. A randomized controlled clinical trial
Source: BMC Oral Health. 2026 May 13;26:881. doi: 10.1186/s12903-026-08534-w (PMC13192118; doi:10.1186/s12903-026-08534-w)
Supplement: Supplementary file 2 — Supplementary Material 2. Additional file 2: Dynamic condyle tracking. [file 12903_2026_8534_MOESM2_ESM.pdf]

AMANN GIRRBACH Artex Report

Person: [redacted]
Record: [redacted] Function and Digital Occlusion, yamany

Person

**First name**	[redacted]
**Last name**	[redacted]
**Gender**	[redacted]

Measurement profile

**Measurement configuration**	Artikulator & Realmovement
**Measurement mode**	Function and Digital Occlusion
**Condyles defined by**	C-Positioner (grey)
**Used bite fork**	REF1960320 Bite fork type SD
**Used attachment**	REF1960250 curved paraocclusal attachment

AMANN GIRRBACH Artex Articulator

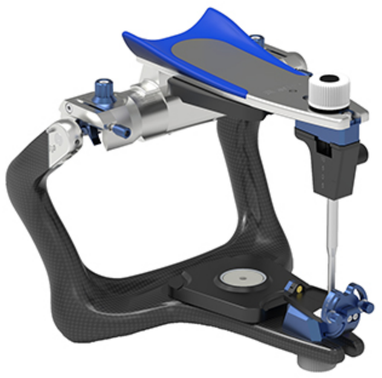

Sagittal Condyle Incl., °	L	17.6	-20 … 60
	R	20.9	
Bennett Angle, °	L	-	0 … 60
	R	10.6	
ISS, mm	L	-	0 … 10
	R	0.1	
Retrusion, mm	L	0.2	0 … 10
	R	-	
Front Table Inclination, °	L	50.0	0 … 80
	R	50.0	
	S	57.6	

AMANN GIRRBACH Artex Report

Person: [redacted]
Record: [redacted] Function and Digital Occlusion, yamany

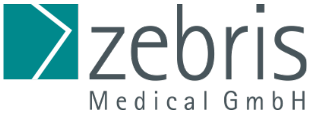

Pin Positions (Artex)

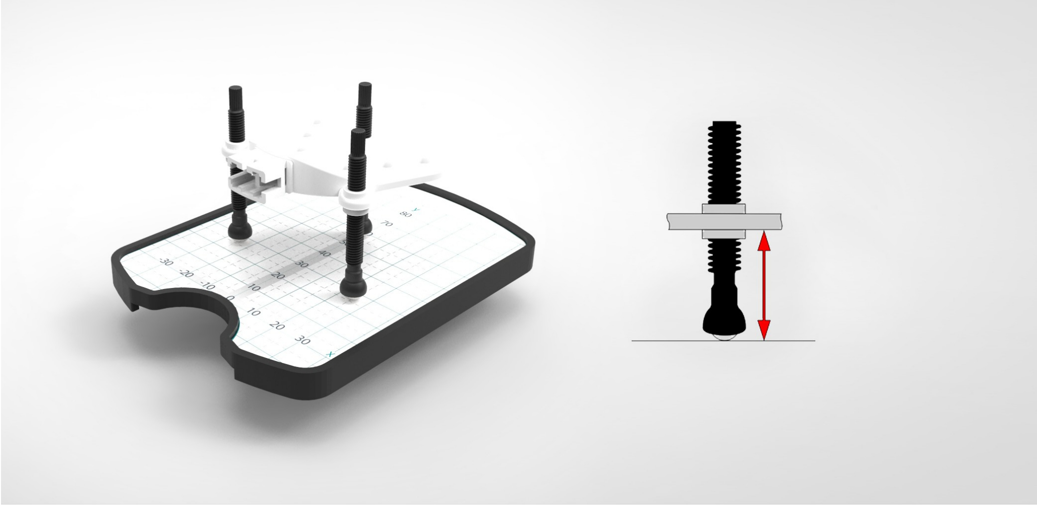

Reference picture

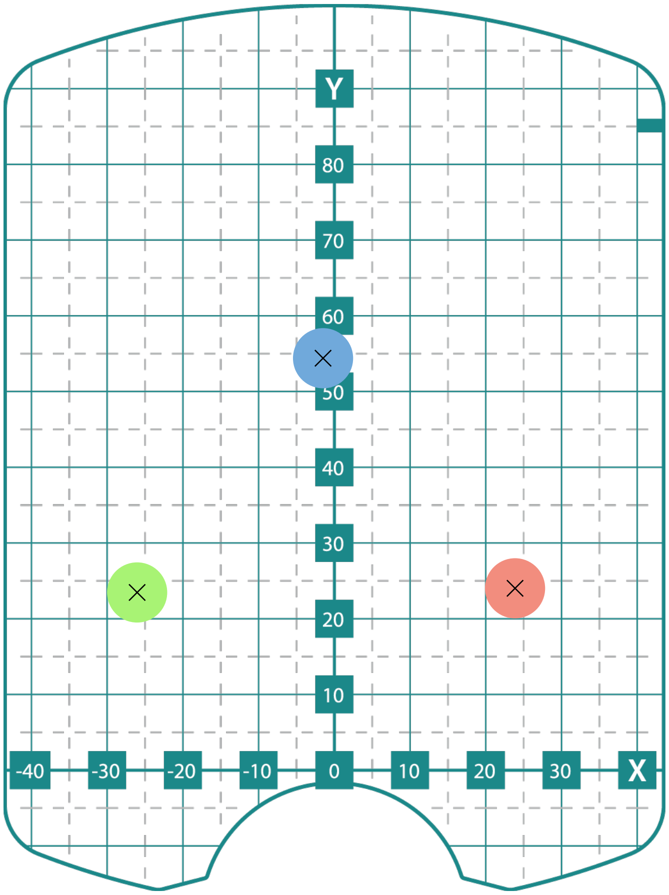

Transfer table values

	Blue	Red	Green
X	-2	24	-26
Y	54	24	23
Pin length	26	22	23

© zebris Medical GmbH

AMANN GIRRBACH Artex Report

Person: [redacted]
Record: [redacted]Function and Digital Occlusion, yamany

zebris Medical GmbH

Tracks (AMANN GIRRBACH Artex)

Sag. Condyle Protrusion, right

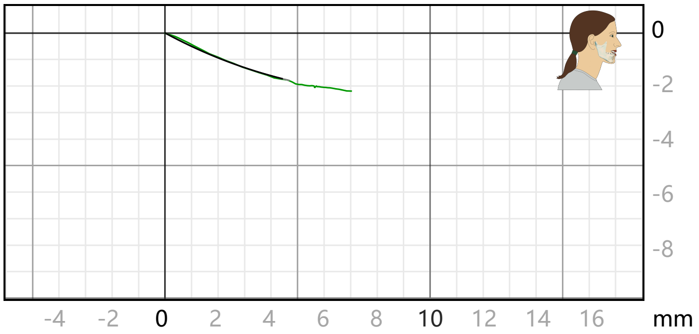

Sag. Condyle Protrusion, left

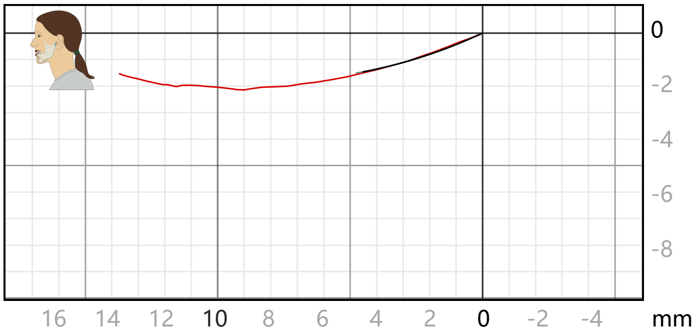

Hor. Condyle Laterotrusion, right

Bennett

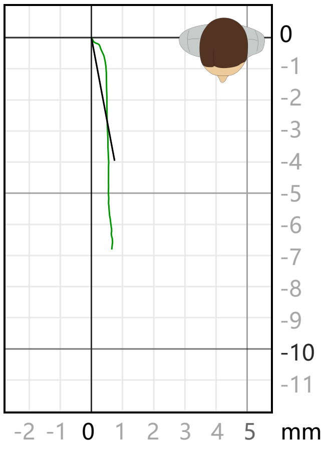

ISS

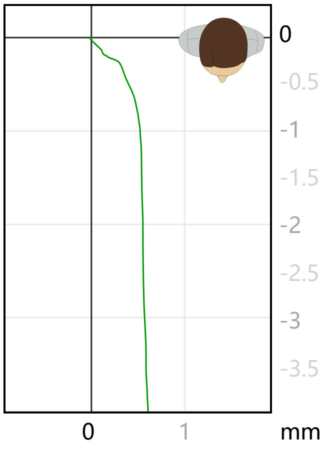

Hor. Condyle Laterotrusion, left

ISS

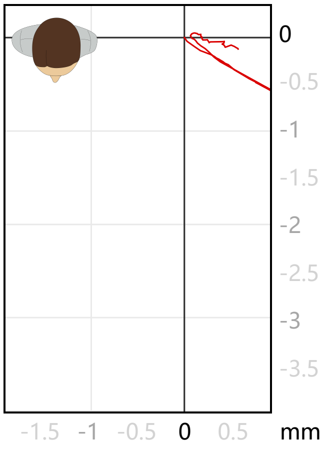

Bennett

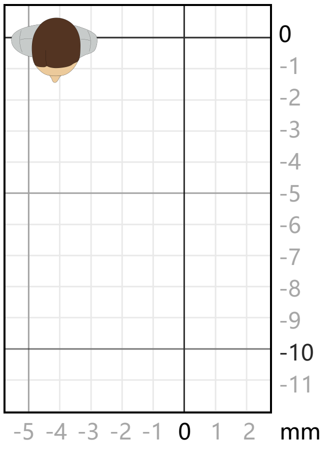

Retrusion

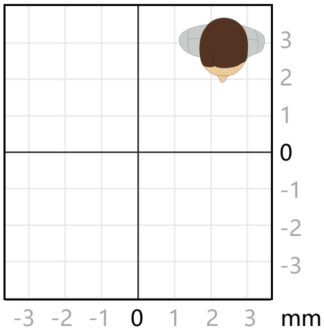

Shift Angle

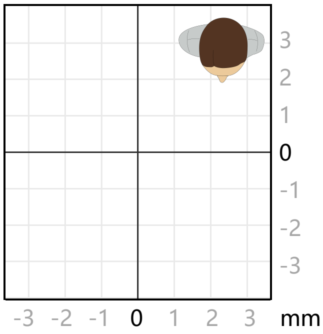

Shift Angle

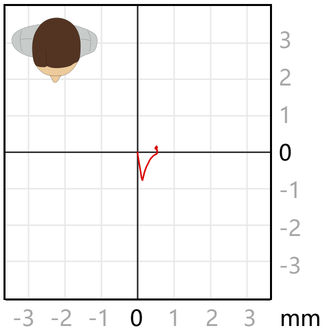

Retrusion

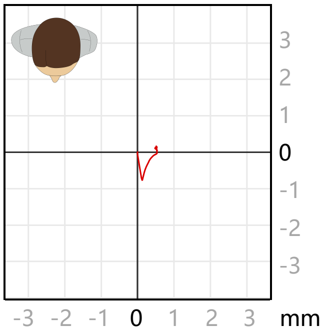

© zebris Medical GmbH

AMANN GIRRBACH Artex Report

Person: [redacted]
Record: [redacted] Function and Digital Occlusion, yamany

zebris
Medical GmbH

Front Table

Protrusion

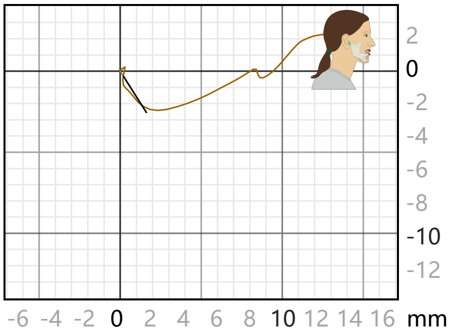

Laterotrusion

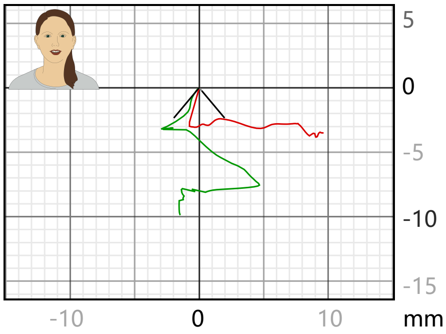

Protrusion and Laterotrusion

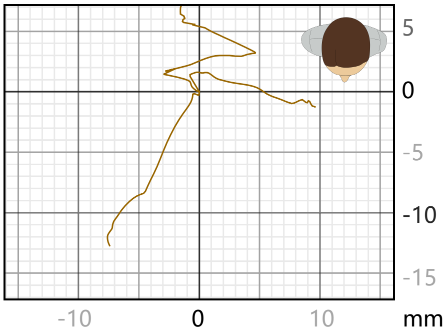

© zebris Medical GmbH
