# Supplementary figures and images for: Fully digital workflow versus conventional methods for fabricating the Michigan appliance in patients with temporomandibular joint disorder. A randomized controlled clinical trial
Source: BMC Oral Health. 2026 May 13;26:881. doi: 10.1186/s12903-026-08534-w (PMC13192118; doi:10.1186/s12903-026-08534-w)

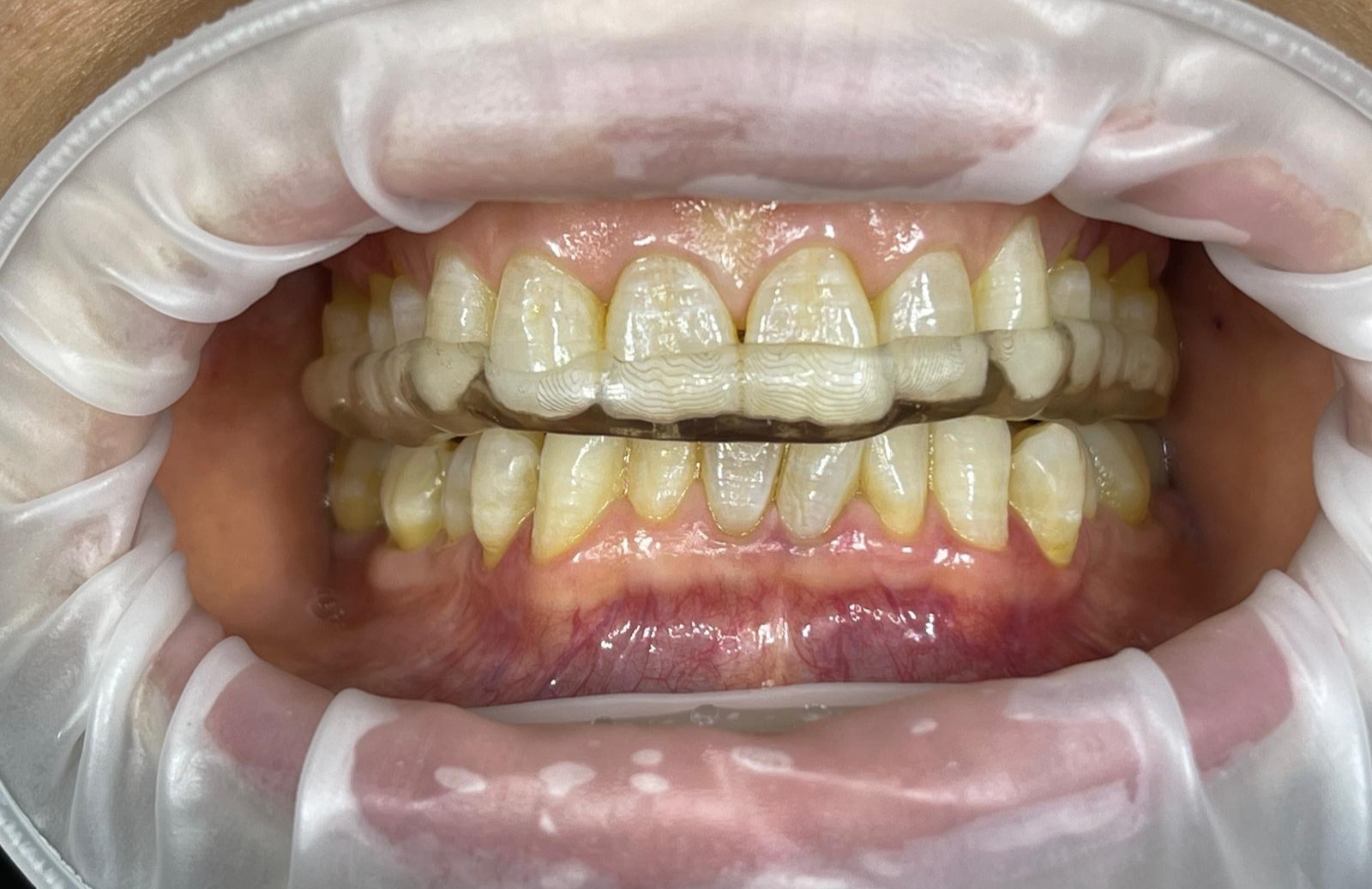

Supplement: Supplementary file 3 — Supplementary Material 3. Additional file 3: A hard acrylic Michigan- splint fabricated using a fully digital workflow. [file 12903_2026_8534_MOESM3_ESM.jpg]
